# A focus on how latent “codons” are unravelled in synthetic copolymers

**DOI:** 10.1039/d3sc90087h

**Published:** 2023-05-23

**Authors:** Christopher Barner-Kowollik, Bryan T. Tuten

**Affiliations:** a School of Chemistry and Physics, Centre for Materials Science, Queensland University of Technology (QUT) 2 George Street Brisbane QLD 4000 Australia christopher.barnerkowollik@qut.edu.au bryan.tuten@qut.edu.au; b Institute of Nanotechnology (INT), Karlsruhe Institute of Technology (KIT) Hermann-von-Helmholtz-Platz 1 76344 Eggenstein-Leopoldshafen Germany christopher.barner-kowollik@kit.edu

## Abstract

We contextualize and highlight an innovative methodology for copolymer analysis introduced by Hibi *et al.* in *Chemical Science* (Y. Hibi, S. Uesaka and M. Naito, *Chem. Sci.*, 2023, https://doi.org/10.1039/D2SC06974A). The authors introduce an advanced mass spectrometric method driven by a learning algorithm, termed ‘reference-free quantitative mass spectrometry’ (RQMS) for decoding sequences of copolymers in real time, including as a function of reaction progress. We highlight potential future implications and applications of the RQMS technique, as well as look forward to where else RQMS could be utilized within the soft matter materials space.

## Abstract 2

We explore and comment on a key development using advanced mass spectrometric methods coupled with advanced algorithmic learning that can assist in analysing complex copolymers.

## Graphical abstract

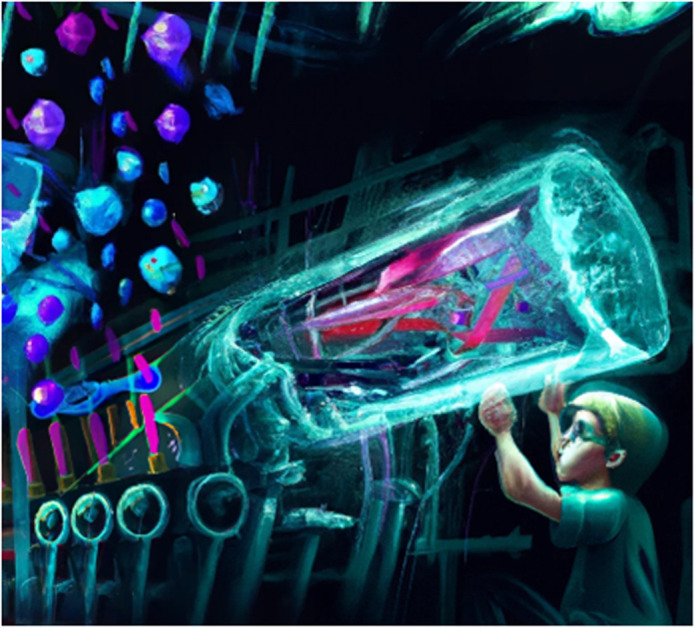

Imagine you have a binary copolymer prepared by free-radical copolymerization at hand and you wish to fully characterize the polymer to understand how its molecular composition relates to its macroscopic properties. In the hands of a polymer chemist, experiments such as size-exclusion chromatography (SEC), differential scanning calorimetry (DSC), thermogravimetric analysis (TGA) and nuclear magnetic resonance (NMR) spectroscopy, as well as mechanical characterization studies, might be employed. In addition, and depending on the molecular weight of the polymer, one might attempt mass spectrometric techniques as well, which in principle provide access to single-chain data. Overall, however, most polymer chemists would concede that a full characterization of this statistical copolymer is impossible, as it is constituted of a wide variety of different chains, each featuring a different number and order of the two monomer units in each chain. Thus, while each individual chain features a defined sequence of monomers in its chain, the overall sample is a complex mixture of such chains.

For most chemists – and of course polymer chemists – this is a fundamentally dissatisfying situation, and thus great effort has been invested into preparing uniform polymers with a defined-sequence, so-called sequence-defined polymers.^1^ However, these tend to be generally harder to prepare than simple free-radical copolymers, for example. Their advantage, however, is that they feature a clear correlation between their structure and their macroscopic properties.

However, even in a complex copolymer mixture prepared by free-radical copolymerization, the resulting macroscopic properties are defined by the chain ensemble constituted by each individual chain. The question is how to identify each chain and its amount within the sample. The current contribution by Hibi, Uesaka and Naito introduces a method by which at least (short) segments of polymer chains within a binary copolymer sample can be identified by mass spectrometric analysis.^2^ If you believe that this sounds like a very complex task, you are correct – after all it is the equivalent of analysing a very complex reaction mixture directly after the reaction without any purification. Welcome to the world of polymer chemistry.

The authors start with the general idea that if one had access to reference materials – in this case, each individual sequence-defined polymer that constitute the overall statistical copolymer assembly – one could deconvolute the observed mass spectrum of the copolymer into its individual chain contributions. While that would indeed be excellent, it is practically impossible as synthesizing each reference chain sample of a statistical copolymer is a task that is beyond even the most advanced organic chemistry laboratory. Instead, the authors introduce a method they call ‘reference-free quantitative mass spectrometry’ (RQMS) to analyse the copolymer sample. RQMS allows access to small sections and the monomer order contained therein of the individual copolymers – not the entire copolymer – which the authors term ‘codons’. While we understand why the authors employ this term (it is borrowed from biochemistry), it is really quite alien to polymer chemists and we recommend it be dropped to align the language of polymer chemistry to the innovation of the authors’ analytical technology. This small semantic matter aside, their method employs a neat trick: instead of making bespoke reference materials, a computer algorithm learns about the short sequences that may be contained within the copolymer to be analysed from similar free-radical copolymers prepared with *varying* amounts of the two (or three) monomer units. The authors note that the larger this ‘basis set’ of reference copolymers is, the longer the sequences in the analyte copolymer that become available should be. They demonstrate that binary triad, binary pentad and ternary triad sequences can be readily identified in statistical polymers, including as a function of the conversion at which the preparation of the statistical copolymers is stopped. Very neat indeed, especially considering – as the authors note – these relatively small segments play a critical role in defining the polymers’ overall properties.

The remaining part of the paper addresses the complex mathematical approaches that are required to conduct the analysis. For the regular polymer chemist, the only question remains: when will user-friendly software be available that will allow us to road test the introduced methodology in our own laboratories?

If a simple user-friendly interface was introduced, the abilities of such a technology could potentially enable a remarkable paradigm-shift in how copolymers are synthesized. With the ability to analyse a polymerization in real-time underpinned by the RQMS methodology introduced by the authors, the future polymer chemist could conceivably adjust the reaction conditions of a copolymerization in real time, in order to bias a desired statistical outcome for the various diad/triad compositions. As alluded to above, in polymer chemistry, there are two ‘schools’ of synthesis: full-sequence definition with high technical difficulty, or virtually no sequence control but low technical difficulty. With very little in between these two methodologies, the ability to impart some control over an otherwise random process underpinned by real-time RQMS monitoring is a highly attractive ‘middle ground’ proposition for polymer synthesis, *i.e.*, allowing for the generation of polymers with sufficient control to impart desired physical properties with minimized technical difficulty.

While radical chain addition was the primary focus of the authors’ work, it would further be interesting to see their methodology applied to a complex mixture of monomers in a step-growth system. Industry often utilizes certain ratios of hard and soft segments in order to achieve their desired physical properties; thus a system capable of informing the chemist what the ratios formed *in situ* are would be a substantial benefit in terms of research and development time.

## Author contributions

Both authors contributed equally to this article.

## Supplementary Material
